# Super Rice With High Sink Activities Has Superior Adaptability to Low Filling Stage Temperature

**DOI:** 10.3389/fpls.2021.729021

**Published:** 2021-10-27

**Authors:** Congshan Xu, Fei Yang, Xinao Tang, Bo Lu, Ziyu Li, Zhenghui Liu, Yanfeng Ding, Chao Ding, Ganghua Li

**Affiliations:** ^1^College of Agronomy, Nanjing Agricultural University, Nanjing, China; ^2^Key Laboratory of Crop Physiology Ecology and Production Management, Ministry of Agriculture, Nanjing, China; ^3^Jiangsu Collaborative Innovation Center for Modern Crop Production, Nanjing, China; ^4^National Engineering and Technology Center for Information Agriculture, Nanjing, China; ^5^Institute of Food Crops, Jiangsu Academy of Agricultural Sciences, Nanjing, China

**Keywords:** super rice, grain filling, temperature, sowing date, sink activity

## Abstract

To investigate the differential responses of super rice grain filling to low filling stage temperature (LT) and the regulative effect of nitrogen panicle fertilizer (NPF), physiological and molecular experiments were conducted with two super rice varieties (Nanjing 7th: N7 and Nanjing 9108th: N9108) on two different filling stage temperature treatments implemented by applying two sowing dates [Normal filling stage temperature (CK): Sowed on May 30, T_mean_ = 24.7°C and low filling stage temperature (LT): Sowed on July 1, T_mean_ = 20.3°C], and two NPF levels (0 and 150 kg N ha^−1^). In this study, LT, NPF, and simultaneous LT and NPF treatments suppressed the grain filling in all varieties with different levels. Under LT or NPF treatments, the reduction of grain weight, seed setting rate, and filling rate were closely associated with suppressed starch biosynthesis rate in inferior seeds, suggesting that reduced starch biosynthesis rate, expression, and activities of enzymes encoded by related genes, Floury endosperm-4 (*FLO4*), Starch branching enzyme-I (*SBE1*), and Starch phosphorylase-L (*PHO-l*), were responsible for the grain filling reduction. Under LT or NPF treatments, significantly higher grain filling rates and lower variance were found in N9108 compared to that in N7, which were closely related to their higher starch biosynthesis ability, related gene expression, and enzymes activities. One of the probable explanations of the grain filling difference was the variation in the relative amount of key regulative hormones, Abscisic acid (ABA) and 1-aminocyclopropane-1-carboxylic acid (ACC). These results raise a possibility that super rice with higher sink activities has superior adaptability to LT and NPF due to their higher sink activities.

## Introduction

Rice (*Oryza sativa* L.) is one of the staple food crops in China. It has made a great contribution to China's national economy (Khush, 2013; National Bureau of Statistics of China, 2015). Rapid population growth and economic development are growing pressures for an increase in food production (Zhang et al., 2007). However, the increase in rice yield has declined since the mid-1980s, as reflected by the sharp drop in the rate of annual yield increase from 2.7% in the 1980s to 1.1% in the 1990s (Horie et al., 2005). To further increase yield, breeding efforts have expanded the yield sink capacity (the maximum size of sink organs to be harvested) mainly by increasing the number of spikelets per panicle (Kato et al., 2007). As a result, cultivars with large panicles or extra-heavy panicle types with numerous spikelets per panicle have become available, such as hybrid rice and “super” rice or “super” hybrid rice in China (Cheng et al., 2007; Peng et al., 2008). However, some past research showed that these varieties can hardly realize their yield potential due to their poor grain filling in the field (Peng et al., 1999; Yang et al., 2002; Ao et al., 2008; Yang, 2010). Different grain filling and yield performance were found, in some super rice varieties, with similar panicle architecture and growth duration, suggesting that the panicle architecture and growth duration were not the major limitations of poor grain filling (Wang et al., 2002; Cheng et al., 2007; Zhang et al., 2007). The sink activity, which is represented by starch synthesis ability, could be the limiting factor of rice grain filling and final yield establishment (Venkateswarlu and Visperas, 1987; Ho, 1988; Liang et al., 2001).

Slow grain-filling rate and low grain weight were proved to be attributed to two major factors: the limitation of carbohydrate supplement and suppression of sink activities (Yang and Zhang, 2010). Previous studies showed that the supply of carbohydrates could be altered by abiotic stress conditions and was also closely related to the sucrose-starch conversion by controlling the concentration of the major substrate of the conversion (Yang and Zhang, 2010). Lower concentrations of soluble carbohydrate in the grain were found in response to abiotic stresses conditions and could be responsible for the final grain weight reduction (Yang and Zhang, 2010). The starch synthesis ability of rice grains was also positively correlated with the grain filling rate, and further controls the final grain weight (Wang et al., 2008; Yang and Zhang, 2010). The starch synthesis can be controlled by several factors, such as Floury endosperm-4 (*FLO4*), Starch branching enzyme-I (*SBE1*), and Starch phosphorylase-L (*PHO-l*) (Yoshida and Hara, 1977; Yang et al., 2001a; Zhao et al., 2004; Zhu et al., 2004; Duan and Sun, 2005). Therefore, the difference in the sink activity of rice grains could be explained by exploring the activity and expression of the target enzyme in the grain filling stage. Previous studies always focused on the gap between superior and inferior seeds of different varieties (Yang et al., 2008). However, the difference in filling rates of superior and inferior seeds is also a major contributor in grain yield and weight establishment.

With the rapid development of agriculture and the change of planting structure, the traditional manual transplanting mode has been replaced by mechanic transplanting (Peng, 2016; Zou and Huang, 2018). This can be observed in the middle and lower reaches of the Yangtze River, known as the major rice-wheat rotation cropping region where lower sowing dates have occurred due to traditional manual transplanting mode being gradually replaced by mechanized transplanting (Xing et al., 2016). As a result, the late sowing date postpones the rice growth duration, which leads to a lower temperature, suppressed rice filling, and reduced grain weight, which consequently produces a reduced grain yield (Qiu et al., 2016). Low temperature stress in the reproductive stage was correlated to the suppression of gene expression related to starch accumulation (Sipaseuth et al., 2007; Jacobs and Pearson, 2010). It was also correlated to further reducing the biosynthesis of starch in different varieties. However, the response of rice, especially “super” rice, and underlying physiological mechanism were still not clear. Sowing time is always given great importance in fields to ensure that rice plants avoid low-temperature stress and in completing the grain filling process earlier (Van Loon et al., 2014). Many previous studies have investigated changes in grain filling in response to different temperatures during the reproductive stage. However, most of those studies were carried out in a controlled greenhouse. Greenhouse studies are unlikely to accurately imitate the long-term cultivation in the field conditions and, therefore, show discrepant results from field studies (Patindol et al., 2015). Some previous studies also adopted this method to mimic low temperature treatment in the field. Wang et al. (2015) performed a field study to investigate the effects of environmental temperature on rice starch accumulation. On the other hand, many previous studies showed that grain filling was mainly controlled by reproductive stage temperature, while light treatment only slightly suppressed the grain filling (Yoshida and Hara, 1977). Thus, we adopted similar sowing dates to mimic the variation of reproductive stage LT stress. The plant hormones that mediate spikelet development, especially ethylene and abscisic acid (ABA), play important roles in regulating grain filling. A higher rate of ethylene evolution in developing seeds suppresses the expression of most starch-synthesis genes and inhibits the activities of starch synthesis-related enzymes which thus leads to a low grain-filling rate (Yang et al., 2006; Zhu et al., 2011; Panda et al., 2018). The 1-aminocyclopropane-1-carboxylic acid (ACC), as the precursor of ethylene biosynthesis, was found to be closely correlated with ethylene content, and could significantly downregulate the grain filling rate (Yang et al., 2006). ABA acts as a sensitive signal during abiotic stress in plants, and its role in grain filling is complicated (Wang et al., 2019). It is notable that ABA plays a key role in grain filling by regulating the sink activity. In addition, it functions in a dose-dependent manner (Wang et al., 2015). An appropriate concentration of ABA can enhance the activities of enzymes involved in sucrose cleaving and starch synthesis and increase the expression of genes related to starch metabolism (Wang et al., 2015). Davies (1995) proposed that plant hormones can act either synergistically or antagonistically and is the balance between promoting and inhibiting agents that ultimately determines the path of plant growth and development. Yang et al. (2006) found that the grain filling rate was not only correlated with the concentration of ABA and ethylene, but also with the ratio of ABA and ACC. In this study, the metabolite content dynamics, ACC and ABA, were also measured to find out their regulative mechanism under low temperature condition.

Nitrogen was always adopted as one of the most important regulators of rice growth, grain yield, and quality (Kirk et al., 1997). Nitrogen panicle fertilizer (NPF) is always necessary for the field conditions to enhance the spikelet number, whereas the seed setting rate was suppressed (Ding et al., 2003, 2010, 2014; Zhang et al., 2007). Some studies showed that rational utilization of nitrogen fertilizer improved the activity of grain starch synthesis (Zhang et al., 2008). Other studies showed poor grain filling and no increase in the number of grains per panicle simultaneously (Mae, 1997; Yang A. et al., 2004; Samonte et al., 2006). Moreover, high nitrogen levels in grains reduced the carbohydrate accumulation in plant source organs, and carbohydrate translocation from source organs to grains would eventually cause poor grain filling (Fu et al., 2019). Unlike in regular conditions, nitrogen fertilizer application could reduce the grain yield in low temperature treatment because of the reduced filling rates (Cao et al., 2018; Jia et al., 2019). Moreover, nitrogen panicle fertilizer has different effects on different cultivars (Fageria and Santos, 2015; Ding et al., 2020). Thus, the differential responses of super rice cultivars and LT in the filling stage to NPF were discovered in this study. Three experimental factors, including filling stage temperatures, NPF levels, and varieties were used in this study to clarify the mechanism of super rice varieties with different sink activities response to LT and NPF, which is critical to the accurate underlying mechanism of the effect of nitrogen fertilizer on super rice varieties with LT condition.

## Materials and Methods

### Experimental Sites

The field experiments were conducted in 2016 and 2017 in the subtropical environment of Danyang City, Jiangsu Province, China (32°0′ N, 119°70′ E, 51 m altitude). Weeds, pests, and diseases were intensively controlled to avoid yield loss. The climate data regarding daily radiation and air temperature were measured at a meteorological station located within 1 km of the experimental site. The daily solar radiation and temperature were measured by a silicon pyranometer (LI-200, LI–COR Inc., Lincoln, NE, USA) and a temperature/RH probe (HMP45C, Vaisala Inc., Helsinki, Finland), respectively. The meteorological data of the two rice growing seasons were shown in Table 1.

**Table 1:** The mean daily temperature (T_mean_, °C), minimum temperature (T_min_, °C), maximum temperature (T_max_, °C), and accumulated temperature (T_accumulated_, °C) in response to CK and LT treatments of 2 varieties (N7 and N9108) in 2016 and 2017.

**Stage**	**Parameters**	**Year**							
		**2016**				**2017**			
		**CK**		**LT**		**CK**		**LT**	
		**N9108**	**N7**	**N9108**	**N7**	**N9108**	**N7**	**N9108**	**N7**
Transplanting	T_mean_ (°C)	23.37	23.37	26.76	26.76	24.25	24.25	27.12	27.12
	T_max_ (°C)	29.32	29.32	30.08	30.08	28.77	28.77	31.95	31.95
	T_min_ (°C)	20.02	20.02	23.99	23.99	19.19	19.19	23.17	23.17
	T_accumulated_ (°C)	253.33	253.33	321.07	321.07	285.01	285.01	342.45	342.45
Tillering	T_mean_ (°C)	26.38	26.98	30.99	31.32	27.67	27.97	31.52	31.54
	T_max_ (°C)	33.98	33.98	33.98	33.98	34	34.22	34.22	34.22
	T_min_ (°C)	20.37	20.37	27.23	27.23	21.29	21.29	28.12	28.12
	T_accumulated_ (°C)	612.33	633.79	573.08	555.12	671.27	718.73	602.42	581.5
Panicle initiation	T_mean_ (°C)	31.51	30.53	27	27.23	30.59	29.64	26.21	26.44
	T_max_ (°C)	35.25	35.25	33.6	33.6	34.22	34.22	32.62	32.62
	T_min_ (°C)	26.52	24.16	22.6	22.6	25.75	23.46	21.94	21.94
	T_accumulated_ (°C)	660.52	670.65	549.35	557.1	638.18	647.97	534.91	542.45
Grain filling	T_mean_ (°C)	23.12	22.89	17.75	19.28	22.12	21.9	16.99	18.45
	T_max_ (°C)	28.48	28.48	28.29	28.29	27.25	27.25	27.07	27.07
	T_min_ (°C)	11.19	1.52	10.5	10.36	11.3	15.86	10.26	10.26
	T_accumulated_ (°C)	794.79	727.12	484.93	501.97	760.56	695.81	464.05	480.35

### Experimental Design

The experiments were randomized in a complete block design with three replications. Two conventional japonica varieties, namely, Nanjing 9108th (N9108) and Ningjing 7th (N7), with two grain filling temperature, namely, LT (implement by setting late sowing date, average temperature = 20.3°C) and CK (implement by setting early sowing date, average temperature = 24.7°C), were used. Two different nitrogen panicle fertilizer (NPF) levels were adopted in this study, namely, no NPF (N0), and 150 kg N ha^−1^ at panicle initiation (N150), and were applied in panicle initiation. The whole experimental field was applied with the same amount of 450 kg P ha^−1^ (Calcium superphosphate) + 150 kg K ha^−1^ at transplanting, and 150 kg K ha–^1^ at panicle initiation stage (Potassium chloride) + 330 kg N ha^−1^ as carbamide at transplanting in 2016 and 2017. The soil properties of the topsoil layer (0–20 cm) before transplanting were measured in both years as follows: 1 kg soil contains 1.25 g total N, 6.8 mg ${\text{NH}}_{4}^{+}$, 0.9 mg ${\text{NO}}_{3}^{-}$, 27.9 mg Olsens-P and 168 mg NH_4_OAc-K, and pH = 6.3. Seedlings were sowed on May 30 and July 1, and machine-transplanted on June 20 (CK) and July 20 (LT) in 2016 and 2017, respectively, with the hill spacing for 14 cm × 30 cm. Machine-transplantation was performed using a rice trans-planter (PZ640, Iseki Agricultural Machinery Co., Ltd., Japan).

### Observations and Measurements

#### Development Stage

The dates of sowing, panicle initiation, heading, and maturity were recorded for determining growth duration. Panicle initiation was defined as the day when 80% of stems in a plot presented a white feathery cone inside the leaf sheath of the rice plant. Heading was the date when 80% of the stems in a plot started anthesis. Maturity was the date when 95% of grains turned yellow.

#### Yield and Yield Components

At panicle initiation, heading, and maturity stages, all the plants within an area of 0.5 m^2^ in each plot were sampled for the growth analysis. After recording the plant height, the numbers of stems (main stems plus tillers), and panicles (when presented), the plant samples were separated into leaves, stems, and panicles. At maturity, the panicles were hand-threshed, and the filled spikelets were separated from the unfilled spikelets by submerging them into tap water. The empty spikelets were separated from the half-filled spikelet by winnowing. Three sub-samples of the filled (30 g), the half-filled (6 g), and the empty (3 g) spikelets were taken to count the spikelets number. The dry weights of the rachis, filled, half-filled, and empty spikelets were measured after oven drying at 70°C to a constant weight. The total dry weight at maturity was the sum of the dry weights of the straw (leaves plus stems), rachis, and filled, half-filled and empty spikelets. The spikelets per panicle (spikelets m^−2^/panicles m^−2^), and grain filling percentage (100 × filled spikelets m^−2^/spikelets m^−2^) were calculated. The grain yield was determined from a 5 m^2^ area in the center of each plot and was adjusted to 14% moisture content.

#### Grain Filling Characteristics

We selected 400 panicles that headed on the same day in each treatment, which were tagged to give an accurate record of the flowering date and the position of the spikelets. Superior grains (located on apical primary branches, SS) that flowered on the first 2 days of anthesis (DAA) and inferior grains (located on proximal secondary branches, IS) that flowered on the last 2 days were separated from the panicles (Chen et al., 2013). Thirty tagged panicles from each plot were sampled at every 5 DAA till 45 DAA. The sampled panicles were divided into three groups (10 panicles each) as three replicates. Then, superior and inferior spikelets were separated from the panicles for RNA extraction and measurement of soluble sugars and starch. All the sampled grains were deshelled and immersed in liquid nitrogen and then kept in a −80°C freezer for further analysis. Further 30 tagged panicles (10 panicles formed a subsample) from each treatment were sampled to measure the dry weight of superior and inferior grains at final harvest. The sampled grains were dried at 70°C to constant weight, dehulled, and weighed.

#### RNA Extraction and qRT-PCR

Total RNA was extracted from 0.1 g of inferior seeds (IS) and superior seeds (SS) of rice plants at 5, 10, 15, and 20 DAA using RNeasy Plant Mini Kit (Qiagen, German) following the method described previously (Wang et al., 2017). First-strand cDNA was synthesized from RNA using the RNAprep Pure (Tiangen, Beijing, China). Relative expression levels of target genes, such as *FLO4, SBE1*, and *PHO-l*, were detected by the Real-time PCR System (Takara, Dalian, China). Primers were designed based on the anticipated size of the amplification products (150–250 bp) as listed in Supplementary Table 1. Primer Premier 5.0 software (Premier, Ottawa, ON, Canada) was used to complete the design work. Three biological replicates were used. Expression levels were normalized to the reference genes: *Actin* (OSNPB_110163100). The primers were designed as shown in Supplementary Table 1.

#### Assays of Grain Weight, Non-structural Soluble Carbohydrates, and Starch Content

To investigate how sowing date affected the grain-filling process, we measured the weight of superior and inferior grains, and the contents of soluble sugars and starch of the inferior grains under the two water supply treatments. A total of 100 SS and IS grains each was used for the measurement of grain dry weight. The samples used for measuring the starch and non-structural soluble carbohydrate (NSC) contents were ground into fine powder, and 500 mg ethanol (v/v) was added. The tubes were kept in a water bath at 80°C for 30 min. After cooling the tubes in water, they were centrifuged at 5,000 × g for 10 min. The supernatant was collected, and the extraction was repeated three times. The sugar extract was then diluted to 50 ml with distilled water and the sucrose content was measured as described by Yang et al. (2001a,c). The residues left in the centrifuge tubes after extracting sugars were dried at 80°C for starch extraction using HClO_4_ following the method described by Yang et al. (2001c). Amylose and amylopectin contents were determined according to He (1985) with minor changes. Amylose content was quantified at 620 and 479 nm, while amylopectin content was quantified at 556 and 737 nm, respectively.

### Metabolite Extraction and Quantification

Fifty milligrams of plant sample was weighed into a 2 ml plastic microtube and frozen in liquid nitrogen, dissolved in 1 ml methanol/water/formic acid solution (15:4:1, v/v/v). Ten microliters of internal standard mixed solution (100 ng/ml) was added into the extract as internal standards for the quantification. The mixture was vortexed for 10 min, and centrifuged for 5 min (12,000 rpm/min, and 4°C), and the supernatant was then transferred to clean plastic microtubes, followed by evaporation to dryness and dissolved in 100 μl 80% methanol (v/v), and filtered through a 0.22 μm membrane filter for further LC-MS/MS analysis. ABA and ACC contents were detected by MetWare (http://www.metware.cn/) based on the AB Sciex QTRAP 6500 LC-MS/MS platform (Wuhan Metware Biotechnology Co., Ltd., Wuhan, China). The quantification of ACC and ABA were analyzed using an UPLC-ESI-MS/MS system (UPLC'ExionLC™ AD' https://sciex.com.cn/; MS'Applied Biosystems 6500 Triple Quadrupole, https://sciex.com.cn/). The content of ACC and ABA was determined using the external standard method and is expressed as ng/g fresh weight (FW). Three biological replications were performed.

### Statistics Analyses

Analysis of variance was performed using SPSS version 20.0 (SPSS Statistics, SPSS Inc., Chicago, USA), and the results are expressed as means (±SD) of three biological replicates. The treatment means were compared based on the least significant difference (LSD) at a 0.05 level of probability.

## Results

### Grain Yield and Yield Components

The present study was carried out to determine the effect of different temperature and sowing dates on the yield and yield related attributes of rice during the rice-growing seasons 2017–18 as presented in Table 1. To accomplish the filling stage temperature treatments in the field, two different sowing date treatments were adopted. Lower Mean temperature (Tmean), Day temperature (Tday), and Night temperature (Tnight) and shorter growth duration were found in LT treatment compared to that in CK treatment (Tables 1, 2). The LT and NPF treatments showed a significant effect on total spikelets number, seed setting rate, and 1,000-seed weight that determine the yield of the rice. The total spikelet number could be enhanced by both NPF and LT treatments while seed setting rate and 1,000-seed weight reduced. Moreover, their balancing eventually altered the grain yield (Table 3). However, the grain yield responds differentially to NPF between CK and LT treatments. Under CK condition, the grain yields of N9108 and N7 could be significantly improved by NPF for about 12.7 and 9.4%, respectively, mainly due to enhanced population size (total spikelet number). However, under LT conditions, the grain yield of both varieties showed no significant change if NPF was applied or not since significantly lower seed-setting rate and 1,000-seed weight were observed, although their total spikelet number increased at the same time. The higher seed-setting rate (12.3 ± 2.4%) and 1,000-seed weight (7.4 ± 1.3%) of both varieties were found in CK treatment compared to those in LT treatment. Similarly, a lower seed-setting rate (9.1 ± 1.7%) of both varieties could be induced by applying NPF.

**Table 2:** Growth duration (dates and days after sowing) of each growth stage in response to CK and LT treatments of two varieties (N7 and N9108) of 2016 and 2017.

**Varieties**	**Treatments**	**Sowing**	**Transplanting**	**Panicle initiation**	**Heading**	**Grain weight stable**	**Mature**
N9108	CK	(5.31) 0	(6.20) 21	(7.27) 58	(8.26) 88	(9.30) 123	(10.30) 153
	LT	(7.10) 0	(7.20) 20	(8.16) 47	(9.17) 79	(10.27) 119	–
N7	CK	(5.31) 0	(6.20) 21	(7.29) 60	(8.30) 92	(10.4) 127	(10.27) 150
	LT	(7.1) 0	(7.20) 20	(8.15) 46	(9.16) 78	(10.21) 113	–

**Table 3:** Yield components of rice varieties (N7 and N9108) in response to fertilizer treatments (N0 and N150) and different filling stage temperatures (CK and LT) in 2016 and 2017.

**Year**	**Varieties**	**Treatments**	**Total spikelets number**	**Seed-setting rate**	**1,000-grain weight**	**Grain yield**
			**(m^**−2**^)**	**(%)**	**(g)**	**(t/ha)**
2017	N9108	N150-CK	48796.8b	85.2ab	30.37a	12.06a
		N0-CK	34233.3c	89.4a	30.50a	9.33c
		N150-LT	53080.3a	76.7c	28.41b	11.42b
		N0-LT	47486.3b	80.8b	29.11ab	10.17b
	N7	N150-CK	51236.7a	75.6ab	27.67ab	10.87a
		N0-CK	40887.7b	82.1a	28.22a	9.47b
		N150-LT	55838.3a	60.1d	27.91ab	9.36b
		N0-LT	52644.0a	66.8c	26.98b	9.20b
2016	N9108	N150-CK	45512.3a	84.1ab	28.86a	11.91a
		N0-CK	27558.7b	90.1a	28.88a	9.23b
		N150-LT	41615.3a	75.1b	27.67ab	9.33b
		N0-LT	44336.5a	82.1ab	26.09b	10.23ab
	N7	N150-CK	55499.0a	78.7b	26.71a	10.93a
		N0-CK	40963.6b	85.6a	25.89b	9.08b
		N150-LT	53182.0a	65.6c	26.36a	9.20b
		N0-LT	46760.4ab	77.3b	26.12ab	9.45b

*Letters after the values indicate statistical significance at the P = 0.05 level*.

### Grain Filling Rate

**Figure 1:**
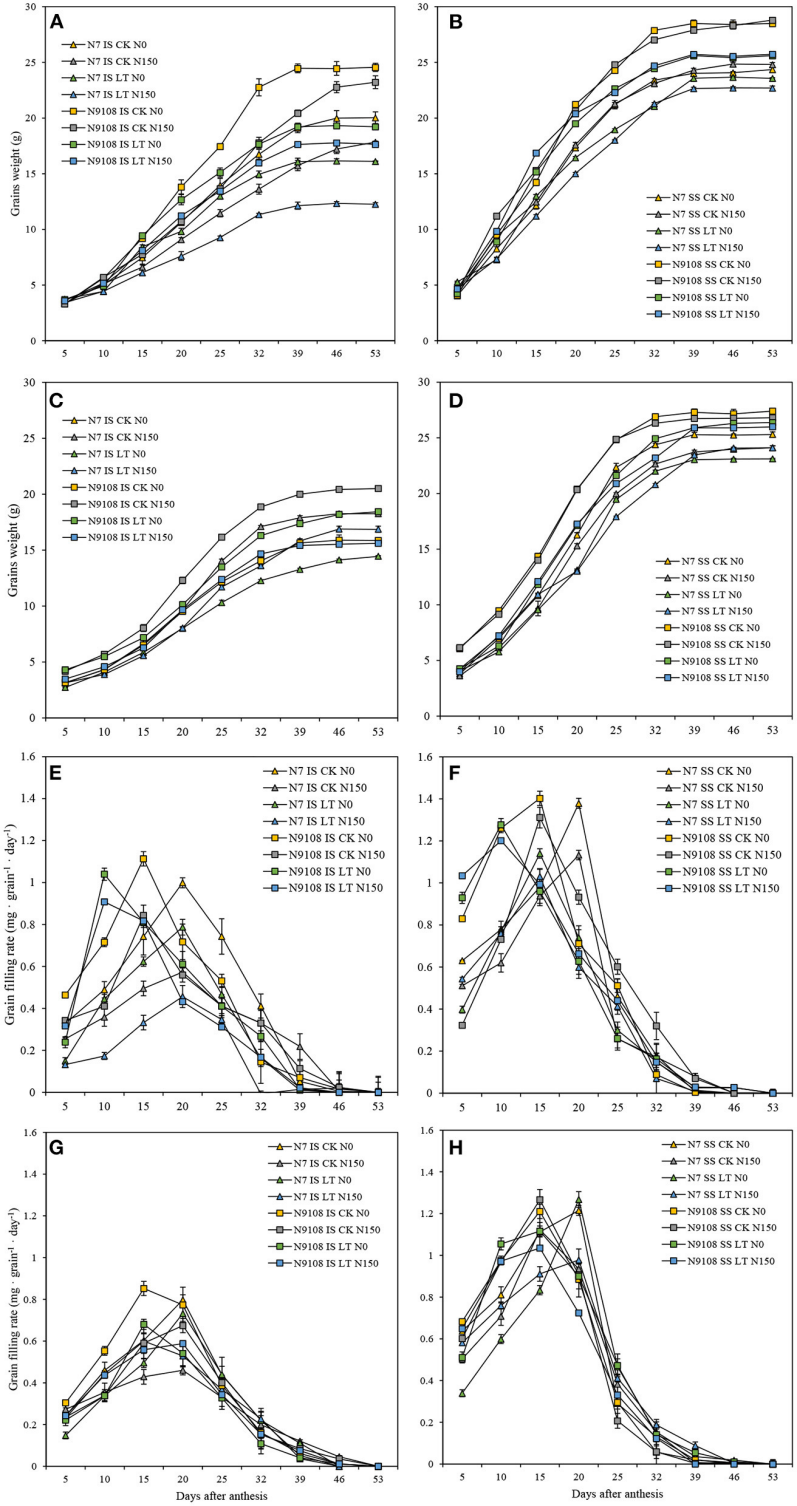
Grain weight and filling rate dynamics of superior (SS) and inferior (IS) in two varieties [Nanjing 7th (N7) and Nanjing 9108th (N9108)] in response to different fertilizer treatments [N150, Applying nitrogen panicle fertilizer (NPF) treatment; N0, No NPF treatment] in filling stage temperature treatments [Normal temperature (CK) and Low temperature (LT) of 2016 and 2017]. **(A)** Grain weight dynamics of IS in 2016; **(B)** Grain weight dynamics of SS in 2016; **(C)** Grain weight dynamics of IS in 2017; **(D)** Grain weight dynamics of SS in 2017. **(E)** Grain filling rate dynamics of IS in 2016; **(F)** Grain filling rate dynamics of SS in 2016; **(G)** Grain filling rate dynamics of IS in 2017; **(H)** Grain filling rate dynamics of SS in 2017.

As shown in Table 4, the grain filling rate of N9108 is significantly higher than that of N7, and it is more significant under the LT condition (Figure 1). Under CK condition, the inferior and superior mean grain filling rate (GR_mean_) of N9108 were 0.504 ± 0.073 and 0.811 ± 0.053 (mg·grain^−1^ · D^−1^), respectively, was about 25 and 43% higher than those of N7 [0.372 ± 0.079 and 0.617 ± 0.033 (mg·grain^−1^ · D^−1^)]. However, under LT condition, the GR_mean_ of inferior and superior grains in N9108 were about 0.416 ± 0.075 and 0.803 ± 0.03 (mg·grain^−1^ · D^−1^), which were significantly higher than those of N7 [0.324 ± 0.069 and 0.595 ± 0.011 (mg·grain^−1^ · D^−1^)], with an increment of about 40 and 67% compared with those of CK condition. In this study, both NPF and LT treatments reduced the grain filling rate of the two varieties, delayed the time to reach the maximum grain filling rate, and increased the initial grain filling potential (Table 4). When NPF was applied, the inferior grain filling rate of N9108 and N7 decreased by about 16 ± 2.7% and 27 ± 8.3%, respectively. The time to reach the maximum filling rate was delayed by about 13 ± 3.3% and 21 ± 7.1%, and the initial grain filling potential decreased by about 13 ± 5.4% and 15 ± 7.8%. In addition, the covariance (CV) of filling rates significantly differed by varieties and seed positions, indicating their differential stability. The CV of GR_mean_, max grain filling rate (GR_max_), and max grain weight (W_max_) in inferior seeds of N7 were 0.33, 0.21, and 0.31, respectively. The CV of GR_mean_, GR_max_, and W_max_ in superior seeds of N7 were 0.12, 0.09, and 0.13, respectively. Differently, the CV of GR_mean_, GR_max_, and W_max_ in inferior seeds of N9108 were 0.22, 0.14, and 0.21, respectively. The CV of GR_mean_, GR_max_, and W_max_ in superior seeds of N9108 were 0.07, 0.04, and 0.04, respectively. It is obvious that the stability of superior and inferior grain filling levels of N9108 was significantly higher than those of N7. The stability of the superior grain filling level of both varieties was higher than that of inferior grain filling, which indicated that grain filling of inferior seeds could be more regulated by NPF and LT treatments.

**Table 4:** Grain filling characteristics of different grain positions (SS and IS) of rice varieties (N7 and N9108) in different fertilizer treatments (N150, applying NPF treatment; N0, no NPF treatment) and temperature treatments (CK and LT).

**Materials**	**Treatment and**	**R_**0**_**	**GR_**max**_**	**T_**max**_**	**W_**max**_**	**GR_**mean**_**	**D**
	**position**		**(mg · grain^**-1**^ · D^**-1**^)**	**(d)**	**(mg · grain^**-1**^)**	**(mg · grain^**-1**^ · D^**-1**^)**	**(d)**
N9108	N150-CK-SS	0.70a	1.5ab	11.58c	28.48a	0.74ab	38.94bc
	N0-CK-SS	0.15d	1.71a	15.04b	28.13a	0.85a	33.08c
	N150-LT-SS	0.43b	1.58a	9.64d	25.49b	0.78ab	32.64c
	N0-LT-SS	0.31c	1.60a	11.98c	26.53b	0.80a	32.05c
	N150-CK-IS	0.20cd	0.72c	22.27a	22.57c	0.36c	56.51a
	N0-CK-IS	0.09e	1.21b	21.66a	24.27bc	0.60b	40.46
	N150-LT-IS	0.31c	0.67c	13.73bc	17.41d	0.34c	56.3a
	N0-LT-IS	0.29c	0.86c	14.05bc	19.17cd	0.43bc	46.68b
N7	N150-CK-SS	0.11ab	1.17b	16.18c	24.74a	0.58b	42.63b
	N0-CK-SS	0.07b	1.51a	22.72a	23.96a	0.75a	31.66c
	N150-LT-SS	0.19a	1.11b	13.45d	22.48ab	0.56b	43.08b
	N0-LT-SS	0.19a	1.09b	14.57cd	21.12b	0.50bc	39.28bc
	N150-CK-IS	0.11ab	0.59d	20.58ab	15.12bc	0.29d	65.21a
	N0-CK-IS	0.08b	0.87bc	22.27a	19.88b	0.44c	46.31b
	N150-LT-IS	0.09b	0.77c	18.83b	13.08c	0.39c	41.86b
	N0-LT-IS	0.07b	0.83bc	19.22b	15.03bc	0.41c	38.29bc

*R_0_, Initial grain filling potential; GR_max_, Maximum grain filling rate; T_max_, Time reaching the maximum grain filling rate; W_max_, Weight of a kernel at the time of maximum grain filling rate; GR_mean_, Mean grain filling rate; D, The time of grain filling reaching 99%*.

*Letters after the value indicate statistical significance at the P = 0.05 level*.

### Starch Accumulation

**Figure 2:**
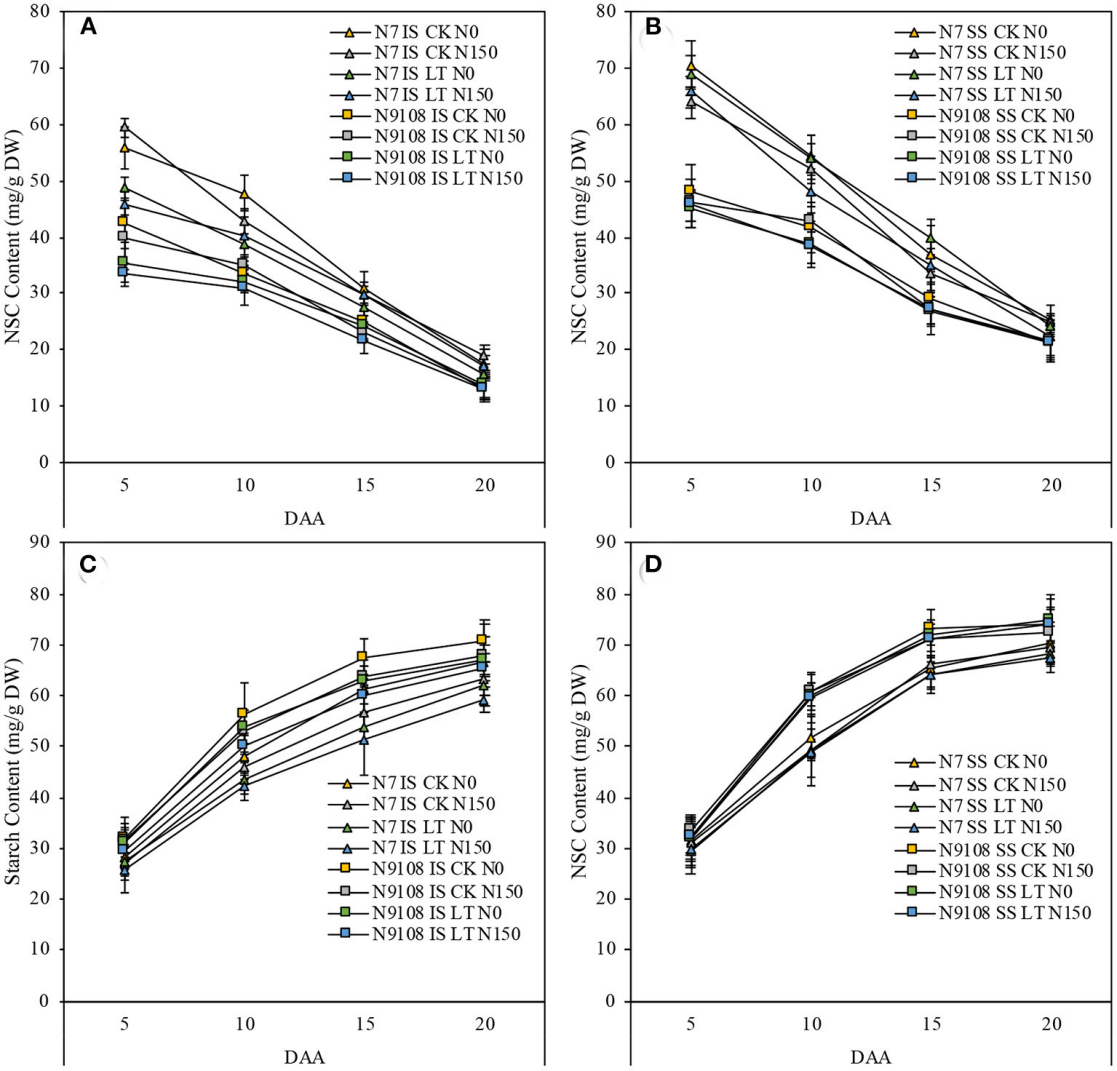
Effect of different nitrogen panicle fertilizer levels (N150: Applying NPF treatment; N0, No NPF treatment) on starch and non-structural soluble carbohydrate (NSC) accumulation dynamics under different filling stage temperature treatments (CK and LT) in IS and SS. DW, dry weight. Values are means (±SD) of three replicates. Samples were taken at 5 days after anthesis (DAA), 10 DAA, 15 DAA, and 20 DAA. **(A)** NSC dynamics of IS in N7 and N9108, respectively; **(B)** NSC dynamics of SS in N7 and N9108, respectively; **(C)** Starch dynamics of IS in N7 and N9108, respectively; **(D)** Starch dynamics of SS in N7 and N9108.

The starch accumulation pattern was consistent with the grain filling rates results as shown in Figure 2. In Figure 2, the starch accumulation of both inferior and superior seeds in N9018 is significantly higher than that in N7. Compared to superior seeds, the starch accumulation and filling rates were significantly higher than that of inferior seeds (Figures 1, 2). Unlike inferior seeds, the starch accumulation of superior seeds was not significantly regulated by temperature and fertilizer treatments. Under CK conditions, the total amount of starch accumulation in inferior seeds was suppressed by NPF. The LT treatment showed a lower inferior seeds starch accumulation compared to that of CK, and the application of NPF had no significant effect under LT conditions. The suppression effect of NPF on starch accumulation in CK condition was higher than that in the LT condition, which was consistent with grain filling results (Figure 2). On the other hand, the effect of temperature and fertilizer treatment on the starch accumulation of N7 was significantly higher than those of N9108 (Figure 2).

**Figure 3:**
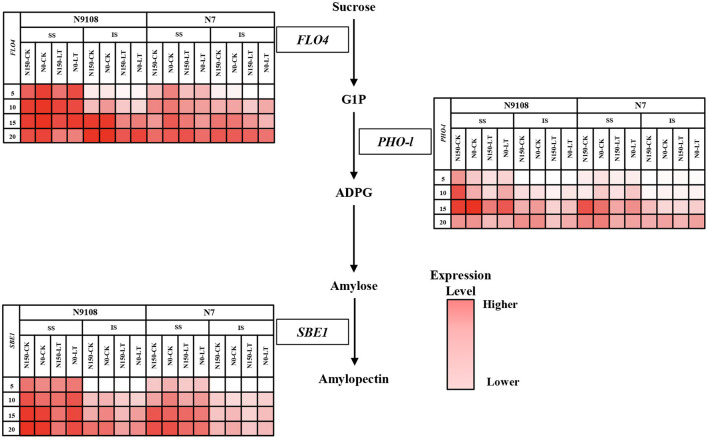
Heat maps of gene expression level involved in the starch biosynthesis pathway. The maps were plotted using the expression level of each gene in the different samples: white indicates low expression level, red indicates high expression level. Two rice varieties (N7 and N9108) were used under different nitrogen panicle fertilizer (N150, Applying nitrogen panicle fertilizer treatment; N0, No nitrogen panicle fertilizer treatment) and subjected to different filling stage temperature treatments (CK and LT). Samples were taken at 5 DAA, 10 DAA, 15 DAA, and 20 DAA. Three genes expression level were measured: *FLO4* (encoding starch synthase), *PHO-l* (encoding starch phosphorylase), and *SBE1* (encoding starch branching enzyme).

To further provide metabolism level clues about changing the biosynthesis of starch, we measured the key intermediate metabolite, amylose, and amylopectin content. We found that, similar to the changing pattern of total starch accumulation, the amylose and amylopectin contents of both varieties were increased rapidly in all measured periods (**Figure 5**). The content of amylose and amylopectin was significantly reduced by LT and NPF treatments in both varieties. On the other hand, the dynamics of amylose and amylopectin were lower in N7 compared to that in N9108, which is consistent with gene expression data (Figure 3).

### Gene Expression

**Figure 4:**
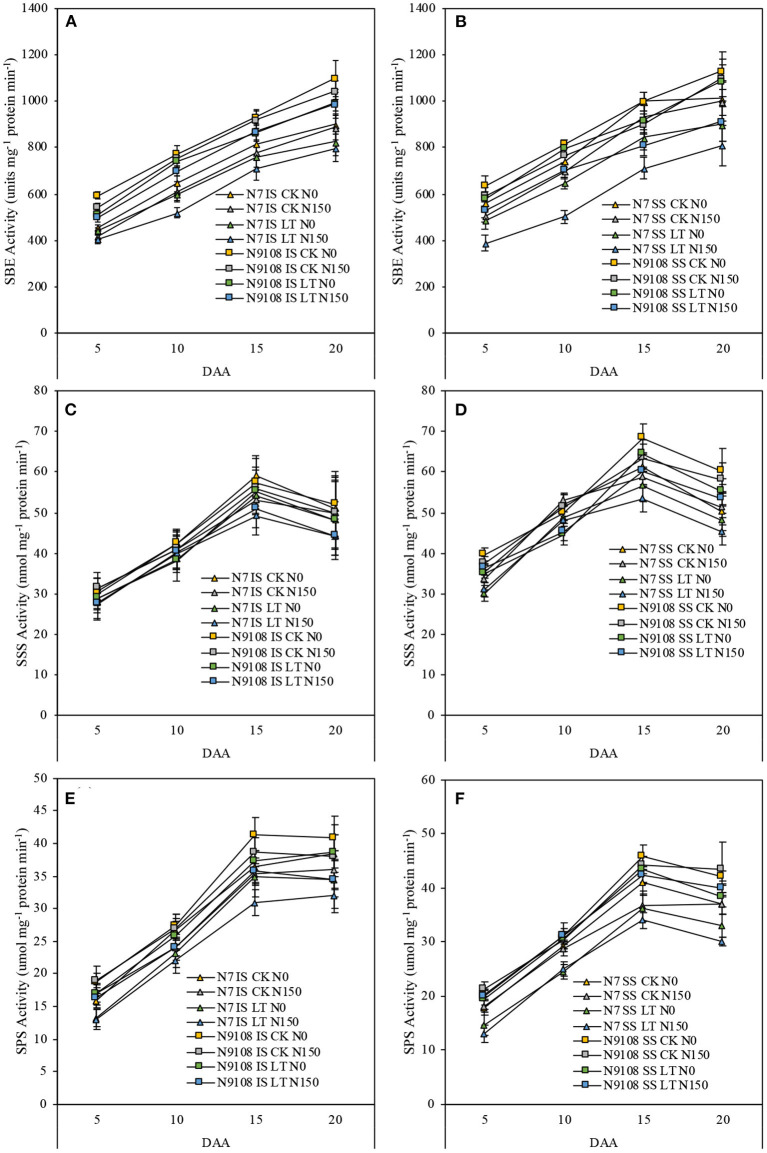
Starch-biosynthesis related enzymes activities (starch branching enzymes (SBE): encoded by *SBE1*, starch synthase (SSS): encoded by *FLO4* and starch phosphorylase (SPS): encoded by *PHO-l*) in IS and SS of two rice varieties (N7 and N9108) under different nitrogen panicle fertilizer (N150, Applying NPF treatment; N0, No NPF treatment) and filling stage temperature treatments (CK and LT). Samples were taken at 5 DAA, 10 DAA, 15 DAA, and 20 DAA Values are means (±SD) of three replicates. **(A)** SBE activities of IS in N7 and N9108, respectively; **(B)** SBE activities of SS in N7 and N9108, respectively; **(C)** SSS activities of IS in N7 and N9108, respectively; **(D)** SSS activities of SS in N7 and N9108, respectively; **(E)** SPS activities of IS in N7 and N9108, respectively; **(F)** SPS activities of SS in N7 and N9108, respectively.

Since the grain filling difference of superior seeds in both varieties among different treatments was not significant, it could be concluded that the difference of grain weight was mainly derived from inferior seeds starch biosynthesis among all treatments. Thus, the relative expression levels of genes related to starch accumulation in inferior seeds were verified in this study (Figure 3). Due to starch, synthesis could be controlled by several key enzymes including starch synthase (SSS, encoded by *FLO4*), starch branching enzyme (SBE, encoded by *SBE* I), and starch phosphorylase (SPS, encoded by *PHO-l*) (Yoshida and Hara, 1977; Yang et al., 2001a; Zhao et al., 2004; Zhu et al., 2004; Duan and Sun, 2005). The expression pattern of the above genes was measured by qRT-PCR and was consistent with the physiological results (Figure 2). *FLO4* gene expression was high and kept stable from 5 DAA (Figure 3). *FLO4* expression, in both varieties under CK condition, was higher than those under LT condition, but there was no significant difference among different NPF application treatments. The expression of *SBE1* in superior and inferior grains increased continuously, while the expression of *PHO-l* in superior and inferior grains increased continuously from 0 to 15 DAA and then decreased slightly since 15 DAA. The expression of *SBE1* and *PHO-l* were significantly suppressed by LT and NPF treatments. Overall, the expression levels of N9108 were significantly higher than those in N7, which indicated a higher starch biosynthesis ability (Figure 3). The gene expression data was consistent with the enzymatic activities data in Figure 4. The encoded gene expression levels and activities of *SBE, SPS*, and *SS* were significantly suppressed by LT and NPF treatments, which also indicated suppressed starch biosynthesis ability.

### Contents of Hormones Related to Grain Filling

**Figure 5:**
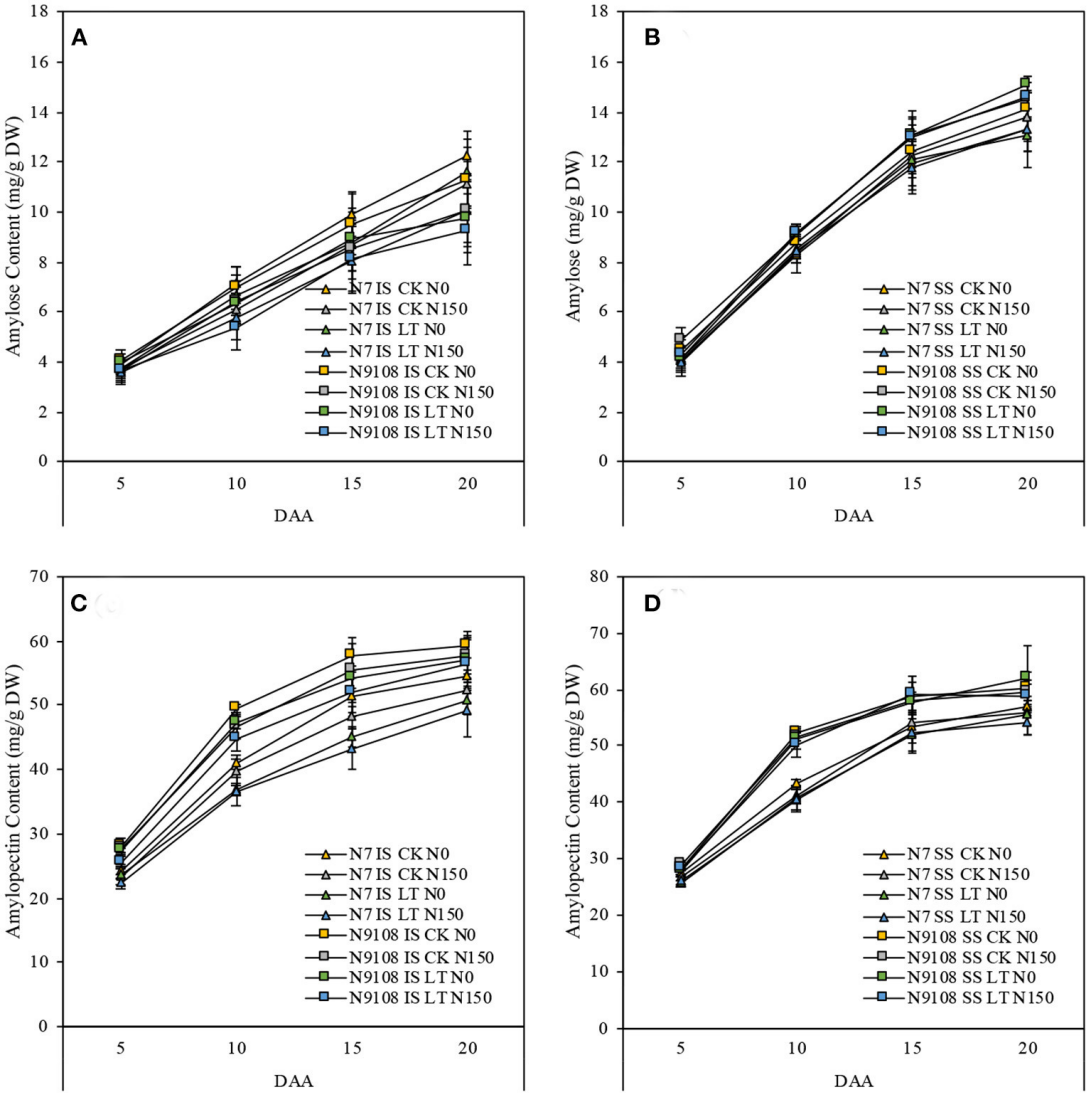
Amylose and amylopectin accumulation dynamics from 5 DAA to 20 DAA in IS and SS of two varieties (N7 and N9108) in response to different fertilizer treatments (N150, Applying nitrogen panicle fertilizer treatment; N0, Without nitrogen panicle fertilizer treatment) and filling stage temperature treatments (CK and LT). Values are means (±SD) of three replicates. **(A)** Amylose content dynamics of IS in N7 and N9108, respectively; **(B)** Amylose content dynamics of SS in N7 and N9108, respectively; **(C)** Amylopectin content dynamics of IS in N7 and N9108, respectively; **(D)** Amylopectin content dynamics of SS in N7 and N9108, respectively.

**Figure 6:**
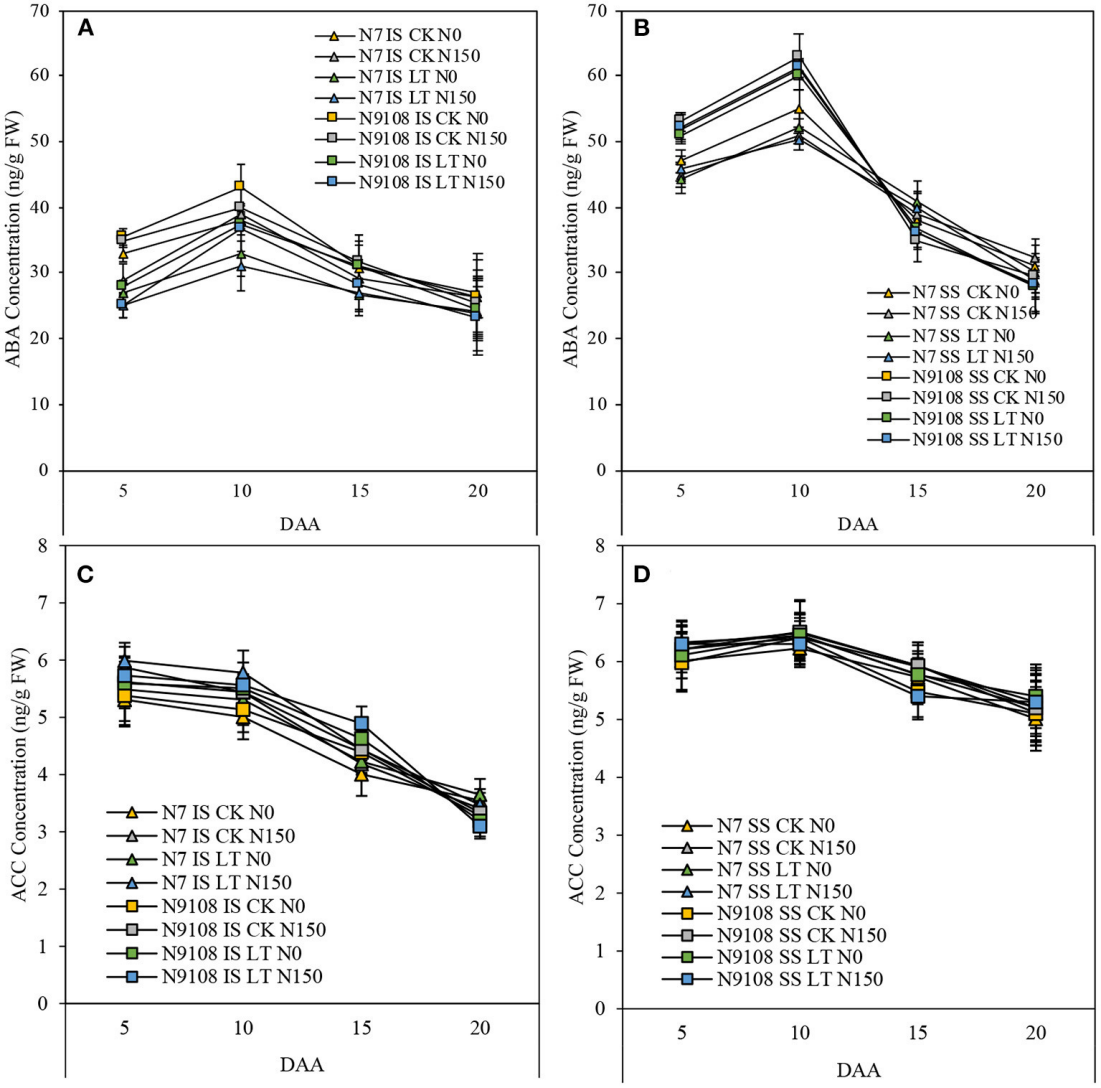
Key hormones related to grain filling such as: Abscisic acid (ABA) and 1-aminocyclopropane-1-carboxylic acid (ACC) in IS and SS of two rice varieties (N7 and N9108) under different nitrogen panicle fertilizer (N150, Applying NPF treatment; N0, without applying NPF treatment) and filling stage temperature treatments (CK and LT). Values are means (±SD) of three replicates. Samples were taken at 5 DAA, 10 DAA, 15 DAA, and 20 DAA. **(A)** ABA concentration dynamics of IS in N7 and N9108, respectively; **(B)** ABA concentration dynamics of SS in N7 and N9108, respectively; **(C)** ACC content dynamics of IS in N7 and N9108, respectively; **(D)** ACC content dynamics of SS in N7 and N9108, respectively.

In this study, we measured the content of ABA and ACC of inferior seeds among different treatments at three grain-filling stages. The ABA concentration was low at the early grain filling stage, increased from 10 DAA, reached a peak at 15 DAA, and declined at 20 DAA. In all treatments, the concentration of ABA was significantly decreased by LT and NPF treatments compared to CK and no NPF treatments, respectively (Figure 5). The reduction was more severe in additive LT and NPF treatment compared to other treatments. In contrast to ABA, the concentration of ACC was high at the early grain filling stage (Figure 6) but continuously declined until 20 DAA. Throughout the grain filling period, the concentration of ACC was largely enhanced by LT and NPF treatments compared to CK and no NPF treatments, respectively. Similar to ABA changing pattern, we found a large increment of ACC concentration in additive LT and NPF treatment compared to other treatments. The variance of ACC responding to NPF was more pronounced than that of LT. The ABA concentration of N9108 among all duration was significantly higher than that of N7, while ACC concentration showed no significant difference.

## Discussion

### NPF and LT Affects Grain Yield

Nitrogen is one of the most important elements for plant growth, and the application of nitrogen fertilizers plays an important role in increasing rice yield due to their increment on total spikelet number (Fu et al., 2019; Wang et al., 2019). However, previous studies also proved that inappropriate nitrogen panicle fertilizer could reduce the grain filling of rice in the field (Ohnishi et al., 1999; Yang A. et al., 2004; Singh et al., 2011; Jiang et al., 2016; Zhang et al., 2021). The grain yield of both varieties could be enhanced by applying NPF since their larger population size. NPF increased grain yield for 11.7 ± 3.3% and 1.6 ± 0.9% under CK and LT conditions, respectively. This meant that the effect of NPF on grain yield was not significant under LT condition (Table 3). Under LT condition, the NPF induced enhancement of the population size and could exaggerate the filling issue similar to how a previous study (Fu et al., 2019) reported. This study explained that under abiotic stress in the filling stage, the grain yield of high nitrogen level was even lower than that of low nitrogen level due to insufficient grain filling. Unlike the responding pattern of the grain yield, both NPF and LT suppressed the grain filling while NPF enhanced grain yield as in previous studies since the increment on total spikelet number (Ding et al., 2003; Wang et al., 2015; Peng, 2016; Fu et al., 2019). Since 20 ± 4.9% higher total spikelet number and 9.7 ± 2.1% lower grain yield was found in N7 compared to those in N9108, it could be concluded that the major difference of the grain yield of the two varieties was mainly derived from the grain weight which was closely related to their filling level instead of their population size. The variance of grain weight of N9108 was significantly lower than that of N7 indicated their higher filling stability (Table 3). To further investigate the underlying mechanism of the different sensitivities of grain weight, the filling rate was examined in this study, and the results were consistent with the grain yield (Table 4).

Abiotic stress, like temperature, suppressed the grain filling rate by slowing enzymes activities, which further reduce the starch biosynthesis (Wang et al., 2021). In the present study, grain filling rate was suppressed by LT but, on the other hand, the late sowing of rice increases the spikelet numbers that are in line with the previous study, which in turn enhances the vegetative stage temperature and rice population size (Wang et al., 2021). As shown in the result, both NPF and LT suppressed the grain filling rate while they both enhanced the spikelet number (Table 3). It could be concluded that increasing grain filling could be a more effective method to increase grain yield in the field rather than increasing population size, since the rice population size has been already enlarged a lot due to the popularization of “super rice” varieties. The grain yield was determined by the balancing between grain filling reduction and spikelet number enhancement. Therefore, the variety selection of late sowing conditions in the middle and lower reaches of the Yangtze River could be guided by strong sink activity rather than sink capacity. The varieties with higher sink activities showed higher LT and NPF adaptivity than low sink activities varieties in this study. The reduction of grain filling level in response to NPF and LT was further proved by lower sugar-to-starch conversion and starch biosynthesis level. Low starch content was found to be produced under LT and NPF conditions, mainly due to lower expression levels of starch biosynthesis correlated with gene expression (*FLO4, PHO-l*, and *SBE1*). A previous study also showed that abiotic stresses affected the grain filling level by controlling starch biosynthesis (Wang et al., 2019). Compared to LT treatment, grain yield could be more significantly regulated by the NPF application in CK condition due to their insufficient filling rate (Table 3). The grain filling rate was reduced by individual LT treatment (15 ± 2.9%). Moreover, the grain filling rate was further reduced (23 ± 4.6%) by applying NPF simultaneously (Table 4 and Figures 2, 3). It could be concluded that higher NPF's yield increasing effect was found in CK condition compared to that in LT condition, which indicated that the appropriate NPF should be selected in LT condition.

#### Sink Activity Controls Rice Grain Filling Rate

The grain-filling issue in inferior spikelets proved to be more serious in the newly bred “super rice” cultivars, although they generally show 8–20% higher yield potential than other conventional rice cultivars due to their large sink size (Kato, 2004; Cheng et al., 2007; Zhang et al., 2007). For example, 12 “super rice” cultivars in the lower Yangtze River basin were investigated in 2006 and 2007, and it was found that the average grain weight and filling proportion of inferior spikelets were 20.9 and 20.7%, respectively, lower than those of superior spikelets, while for three conventional cultivars, on average, it was only 10.5 and 6.3%, respectively. In this study, analyzing the balance between the positive and negative effects of NPF and LT on yield formation indicated that their negative effect on grain weight formation could be more serious than the reduction of grain yield (Table 4 and Figure 1). Thus, we further investigated the grain filling rate among all treatments (Figure 1 and Table 4). The major limitation factor of different grain-filling of super rice varieties with similar sink capacity was assumed as their differential sink activities and carbohydrate supply (Yang, 2010; Yang and Zhang, 2010). In this study, significantly lower sucrose to starch conversion was found in both LT and NPF treatments, which means that sink activity could be the major controlling factor of rice grain weight (Figure 2), similar to a previous study (Yoshida, 1972; Kato and Takeda, 1996; Liang et al., 2001; Ishimaru et al., 2003; Yang A. et al., 2004). The sink activity could be represented by the starch biosynthesis efficiency in rice grains (Kato et al., 2007). In this study, the reduction of grain filling level in response to NPF or LT treatments were only found in the inferior seed rather than in both inferior and superior seeds, similar to the result of Wang et al. (2019). However, unlike that of environmental factors (filling stage temperature or fertilizer), the major controlling factor of grain weight between different varieties was their grain filling rate of both inferior and superior (Table 4). The grain filling rates of N9108 with superior and inferior grains were 15 ± 4.3 and 33 ± 11.6% faster than that of N7, respectively, due to its higher sink activity. Unlike previous study, higher gene expression and starch accumulation was found in both inferior and superior seeds of N7 compared to that of N9108 (Table 4 and Figures 2, 3) which is closely related to higher filling rate and weight in N9108 (Table 4). Therefore, it can be concluded that (1) The sink activities of superior and inferior seeds are the major limiting factors among different varieties' grain filling levels, and (2) the environmental factors (like NPF and LT in this study) mainly affects inferior seeds.

#### NPF and LT Suppressed Grain Filling Related Characteristics

In the process of starch accumulation, more than 30 major enzymes participate in the metabolism of carbohydrates during endosperm development in rice. Among them, several enzymes play important roles in this process, namely, ADP-glucose pyrophosphorylase, granule bound starch synthetase, soluble starch synthase, starch branching enzyme, and starch phosphorylase (Yang et al., 2001a,b; Yang J. et al., 2004; Hannah and James, 2008; Chen and Bao, 2017). The activities of these enzymes are closely related to total starch, amylose, and amylopectin accumulation in rice endosperm. To clarify the underlying regulating molecular mechanism, the genes expression of *FLO4, SBE1*, and *PHO-l* were measured and demonstrated as heatmap in this study (Figure 3). Enzymes involved in starch accumulation are not only affected by genotype but also by the growing environment of rice (Yang, 2001; Yang J. et al., 2004; Halford et al., 2015; Mayer et al., 2016). Many previous studies found that abiotic factors could affect enzyme activity and subsequently, change total starch, amylose, and amylopectin contents (Pan, 1999; Sun et al., 2018; Cheng et al., 2019; Prathap et al., 2019). Among these factors, enzyme activities are sensitive to environmental factors (like nitrogen fertilizer and temperature), and they can, consequently, affect starch accumulation (Cao et al., 2015; Fu et al., 2019), which is consistent with our results in which the gene expression was suppressed by LT and NPF (Figure 3). In this study, the lower gene expression of enzymes related to starch biosynthesis under NPF and LT treatments further reduced the amount of starch synthesis (Figures 1–3 and Table 4) which resulted in the reduced grain yield of both varieties (Table 3). Application of individual NPF and LT treatments decreased the inferior grain weight for about 13 and 21%, respectively, of both varieties, which is similar to previous studies (Mae, 1997; Samonte et al., 2006), while synergistic NPF and LT treatment decreased the inferior grain weight for about 27% (Table 3). The additive and negative effects of NPF and LT treatments could be concluded based on the above results.

Many studies have demonstrated that hormonal changes at the whole-plant level can regulate senescence and nutrient remobilization (Davies, 1995; Lee and Masclaux-Daubresse, 2021). Abiotic stresses also affect hormonal levels, potentially regulating seed development and nutrient mobility (Ober et al., 1991; Davies, 1995; Wang et al., 2006). To further investigate the variation of grain filling and related characteristics, regulative phytohormones content was measured (Figure 6). Ethylene and ABA are two of the major phytohormones induced in response to stress, and their content showed complex changing pattern to biotic and abiotic stresses (Davies and Zhang, 1991; Gazzarrini and Mccourt, 2001; Yang et al., 2001b, 2006; Davies et al., 2002; Wilkinson and Davies, 2002; Cheng and Lur, 2010). It was found that activities of three key enzymes involved in the sucrose-to-starch pathway in the grains. SuSase, AGPase, and SSSase (Hawker and Jenner, 1993; Ahmadi and Baker, 2001; Hurkman et al., 2003), were significantly enhanced by the application of ABA, while ethylene played an inhibitive role in grain filling, since ethylene proved to be a negative regulator of ABA action in the seed (Ghassemian et al., 2000). Many previous studies have also described the interaction between ABA and ethylene in inferior seeds and the relationship between this interaction and starch accumulation pathway (Mohapatra et al., 1993; Kato et al., 2007; Zhu et al., 2011; Zhang et al., 2012; Wang et al., 2015). It was proved by Yang et al. (2006) that higher ABA concentration and lower ACC concentration could improve the grain-filling. In this study, we measured the concentration of ABA and ACC and found that the concentration of ABA was reduced by LT and NPF, while ACC was mainly enhanced by NPF. The result showed that the ratio of ABA and ACC was reduced by LT and NPF, which plays a negatively regulative role in the grain filling process and is consistent with grain filling changing pattern (Figure 6). One of the probable explanations of reduced grain filling in this study is the ratio of ABA and ACC. However, the biosynthesis and catabolism of ABA and ethylene in responding to LT and NPF are still unclear. Hence, the molecular studies on the hormones are necessary to dissect the underlying mechanism.

## Conclusion

The result led to the conclusion that higher sink activities of super rice, namely, higher grain filling rates, which was regulated by the elevated ratio of ABA and ACC, are the major contributor of higher adaptivity to NPF and LT conditions. Moreover, the negative and additive effect of LT and NPF was also found on grain filling events of both varieties. Higher sensitivities of grain filling in response to NPF were found under LT compared to that under CK. This study provides basic knowledge about the mechanism of grain filling of different super rice cultivars with different sink activities in response to LT and NPF.

## Data Availability Statement

The original contributions presented in the study are included in the article/Supplementary Material, further inquiries can be directed to the corresponding author/s.

## Author Contributions

GL contributed to conception and design of the study. CX performed organization of the database, statistical analysis, and manuscript writing. All authors contributed to manuscript revision, read, and approved the submitted version.

## Funding

This work was supported by the National Key Research and Development Program of China (2018YFD0300803, 2017YFD0300100, and 2017YFD0301204), Key Research and Development Program of Jiangsu Province (BE2017369), and the Jiangsu Agriculture Science and Technology Innovation Fund [CX(18)1002].

## Conflict of Interest

The authors declare that the research was conducted in the absence of any commercial or financial relationships that could be construed as a potential conflict of interest.

## Publisher's Note

All claims expressed in this article are solely those of the authors and do not necessarily represent those of their affiliated organizations, or those of the publisher, the editors and the reviewers. Any product that may be evaluated in this article, or claim that may be made by its manufacturer, is not guaranteed or endorsed by the publisher.
