# Supplementary material for: Super Rice With High Sink Activities Has Superior Adaptability to Low Filling Stage Temperature
Source: Front Plant Sci. 2021 Oct 27;12:729021. doi: 10.3389/fpls.2021.729021 (PMC8578116; doi:10.3389/fpls.2021.729021)
Supplement: Supplementary file 1 [file Table_1.docx]

Supplementary Table 1. Specific primers for each starch synthesis related enzyme genes. The primer pairs were designed using the Beacon Designer 7.0 software. The primers used in qRT-PCR are listed in Table. Gene expression was assessed using a Mastercycler EP Realplex RT-PCR system (Eppendorf, https://www.eppendorf.com) and Bestar™ Real-time PCR Master Mix SYBR Green (DBI Bioscience, http://www.xinghanbio.com).

| Gene | Gene ID | Forward sequence (5’-3’) | Reverse sequence (5’-3’) |
| --- | --- | --- | --- |
| *FLO4* | 9268758 | CATGCACTGTTCGAGGAGAA | GGGAAATGGCTCTCCCTTAG |
| *OsPho-L* | 100170240 | TTGGCAGGAAGGTTTCGCT | CGAAGCCTGAAGTGAACTTGCT |
| *OsSBE-1* | 4342117 | TGGCCATGGAAGAGTTGGC | CAGAAGCAACTGCTCCACC |
